# Lung‐Cardiovascular Interactions and Derangements in ARDS

**DOI:** 10.1002/cph4.70254

**Published:** 2026-09-08

**Authors:** Pablo A. Sanchez, Connor O'Brien, Matthieu Legrand, Carolyn S. Calfee, Michael A. Matthay

**Affiliations:** ^1^ Division of Cardiology, Department of Medicine University of California San Francisco San Francisco California USA; ^2^ Department of Anesthesiology University of California San Francisco San Francisco California USA; ^3^ Division of Pulmonary, Critical Care, Allergy & Sleep Medicine, Department of Medicine University of California San Francisco San Francisco California USA

**Keywords:** acute respiratory distress syndrome (ARDS), microcirculation, pulmonary vascular, right ventricle, systemic vascular

## Abstract

The acute respiratory distress syndrome (ARDS) is fundamentally characterized by alveolar injury, leading to lung edema, severe hypoxemia, and poorly compliant lungs. Alveolar and cardiovascular pathology are deeply intertwined in the syndrome, which can be marked by clinical cardiac, pulmonary, and systemic vascular abnormalities. Pulmonary vascular dysfunction is common even in the era of lung‐protective ventilation, is associated with right ventricular (RV) dysfunction, and worse mortality. Frustratingly, these cardiovascular abnormalities pose management challenges for which we have limited targeted therapies that improve clinical outcomes. Recent inroads disentangling patient heterogeneity in ARDS may offer a direct avenue connecting biological mechanisms to effective treatments. Throughout this review, we will examine the clinical involvement of the cardiovascular system in ARDS, grounded on pathological and biological studies, and review available evidence for potential targeted therapies.

## Graphical abstract

This review proposes an anatomical near‐ and far‐field framework for lung‐cardiovascular interactions in ARDS. Grounded in pathological and biological studies, the evidence spans pulmonary vascular, right ventricular, and systemic endothelial derangements and reviews available evidence for targeted therapies.

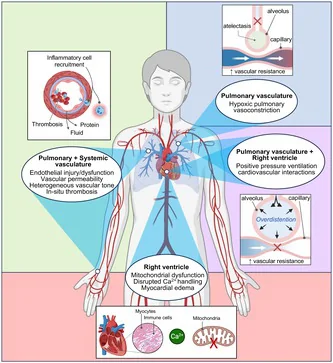

## 1 Introduction

The acute respiratory distress syndrome (ARDS) is defined by the acute development of hypoxic respiratory failure, not explained by cardiogenic pulmonary edema, due to lung inflammation and injury from a specified trigger (Matthay et al. 2024). The syndrome continues to carry a recalcitrantly high mortality rate, despite being first described more than 50 years ago, and millions of dollars spent in research and clinical trials (Bellani et al. 2016). The physiological effects of the syndrome are widespread, ensnaring multiple organ systems. Notably, its prognosis often relies more on extra‐pulmonary organ involvement than on respiratory failure itself (Matthay et al. 2019; Evrard et al. 2024; Sullivan et al. 2025).

The pathogenesis of ARDS is driven by alveolar epithelial and endothelial injury, leading to increased vascular permeability, alveolar accumulation of protein‐rich fluid, neutrophils and red blood cells (Matthay et al. 2019). The alveolar epithelial injury in patients with severe ARDS is extensive, involving focal cell destruction and denudation of the alveolar basement membrane (Matthay et al. 2019; Bachofen and Weibel 1982). Conversely, the endothelial effects are visually more subtle and result from structural and functional changes that disrupt fluid and protein flux across capillaries (Matthay et al. 2019). This endothelial cell activation can occur after direct, nearby, epithelial cell injury by various agents (e.g., viral or bacterial pathogens, aspiration, or hyperoxia), remote inflammatory signaling in non‐pulmonary sepsis leading to lung white blood cell infiltration, blood product transfusions or ischemia–reperfusion (Matthay et al. 2012). This emphasizes that the vascular pathology in the lungs results from a systemic inflammatory response and can involve systemic vascular beds as well, particularly in sepsis‐induced ARDS (Matthay et al. 2019; Wick et al. 2024).

The heart and vascular system are specifically and intimately involved in ARDS pathophysiology. Every patient has abnormalities along the pulmonary vasculature (Tomashefski et al. 1983; Bull et al. 2010), with prevalent systemic vascular dysfunction and cardiac dysfunction (Zochios et al. 2017; Matthay et al. 2019). This multipronged disarray evolves throughout the course of illness, requires careful diagnostic considerations, and calls for nuanced management decisions for which we have limited targeted therapies. Cardiovascular involvement may also play a role in the maintenance of ARDS pathobiology (Ryan et al. 2014) and thus may serve as an avenue for intervention.

**FIGURE 1:**
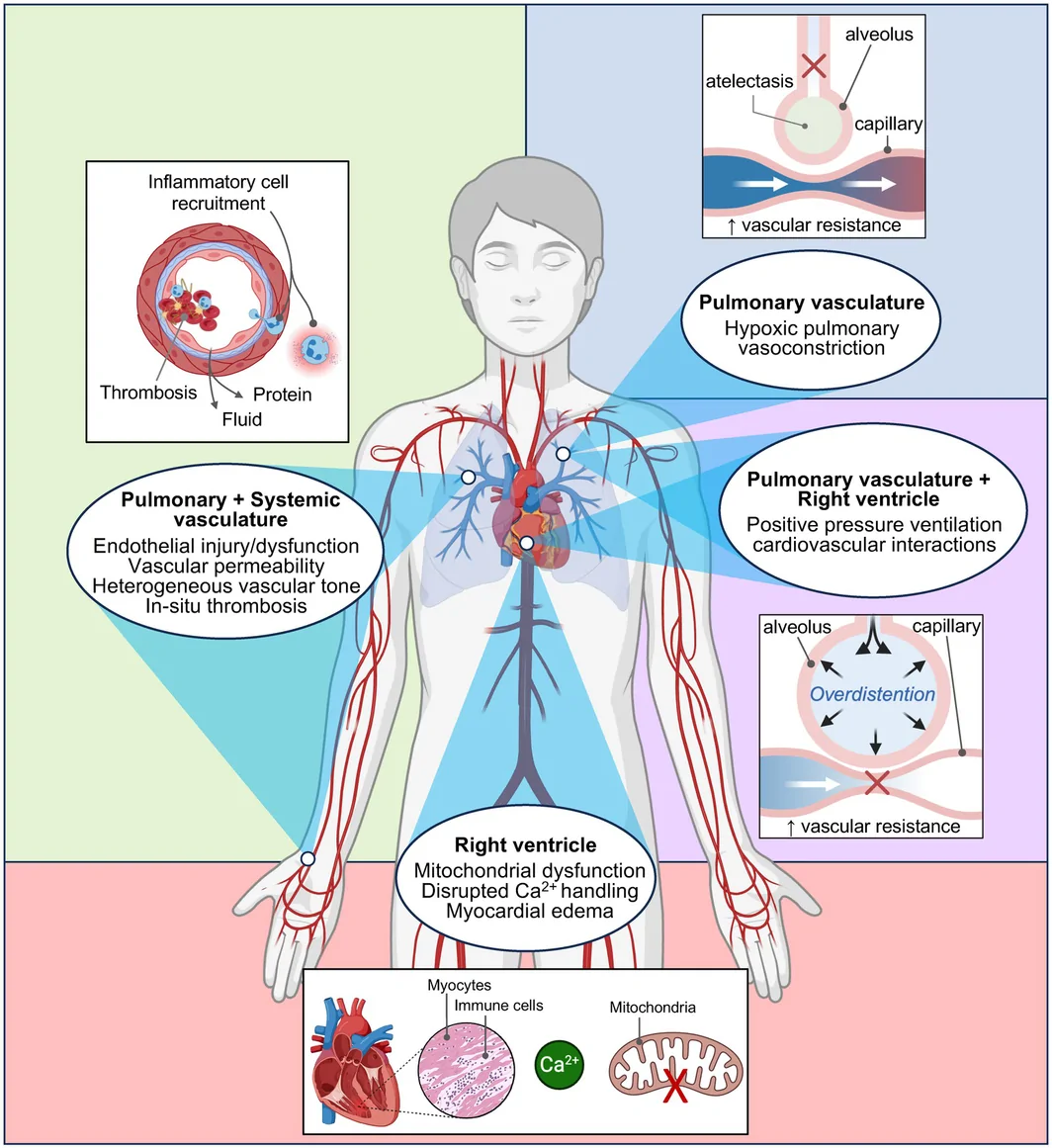
Key mechanisms of lung‐cardiovascular interactions near and far from the lung in ARDS.

This review will explore the relationships between the lung and the cardiovascular system in ARDS. It is organized into the anatomical “near” and “far” fields from the lung (Figure 1), each with sub‐sections reviewing the pathophysiology, clinical manifestations, and available evidence for management.

### 1.1 The Anatomical Near‐Field of the Lung: Pulmonary Cardio‐Vascular Interactions

#### 1.1.1 Pulmonary Vasculature

##### 1.1.1.1 Pathophysiology

Shortly after the first description of ARDS in adults (Ashbaugh et al. 1967), seminal physiology and autopsy reports connected pulmonary vascular pathology to the alveolar epithelial damage (Tomashefski et al. 1983; Snow et al. 1982). The vascular abnormalities in their subsequent autopsy series (Tomashefski et al. 1983), describing early through late stages, involved capillaries and arteries, with endothelial injury and swelling on histological and electron microscopy. Particularly in intermediate and late stages, significant loss of capillary density was seen (Tomashefski et al. 1983). Careful physiologic and animal studies implicate several mechanisms in pulmonary vascular dysfunction including endothelial dysfunction, in situ thrombosis, and vascular occlusion, increased vascular tone, and hypoxic pulmonary vasoconstriction (Price et al. 2012).

**FIGURE 2:**
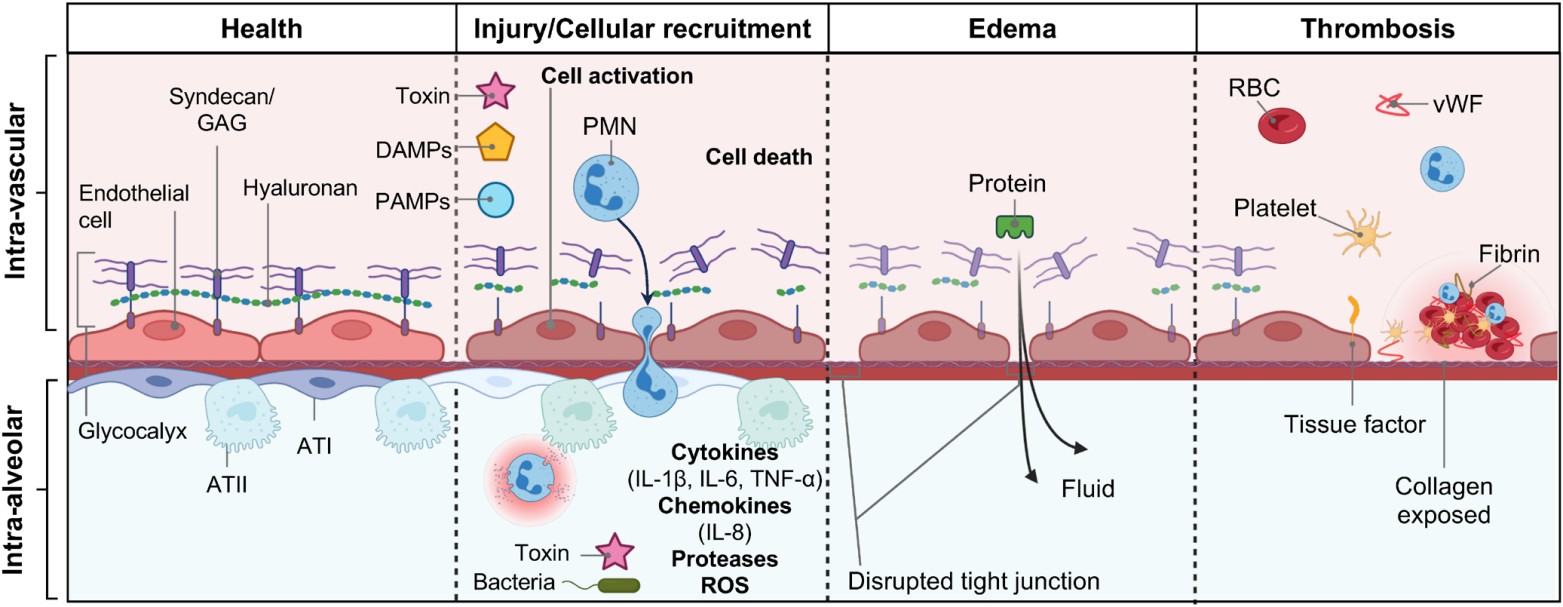
Main mechanisms of vascular pathology in ARDS. In health, the pulmonary and systemic vascular endothelial cell surface is covered, protected and regulated, by the glycocalyx (made up of glycoconjugates and glycans). The alveolar epithelium is composed of alveolar type I (ATI) and type II cells (ATII) which facilitate gas exchange and produce surfactant, respectively. These cell layers are separated by interstitium, and flux of protein and fluid between them is regulated by cell structure and tight junctions. Toxins, DAMPs, PAMPs, or pathogens at the endothelial or epithelial surface lead to degradation of the endothelial glycocalyx, damage and activation of endothelial cells and death of epithelial alveolar cells. Inflammatory pathways lead to the recruitment and migration of leukocytes across the endothelium. Migrated neutrophils release inflammatory mediators, proteases and reactive oxygen species (ROS) which compound the inflammatory response and lead to further injury. Disruption of endothelial tight junctions and endothelial structural changes allow the influx of protein‐rich edema into the alveoli. Tissue factor on activated endothelial cells as well as exposed sub‐endothelial collagen serve as niduses for in situ thrombosis, which can be extensive. DAMPs, Damage‐associated molecular patterns; GAG, glycosaminoglycans; IL, interleukin; PAMPs, Pattern‐associated molecular patterns; PMN, polymorphonuclear cell; RBC, red blood cell; TNF, tumor necrosis factor; vWF, Von‐Willebrand Factor.

Endothelial injury initiates a complex cascade of signaling that affects pulmonary vascular dysfunction and permeability (Figure 2, Table 1). Plasma concentrations of Von Willebrand Factor (which aggregates circulating platelets) (Sabharwal et al. 1995), angiopoietin‐2 (destabilizes endothelial structure), and angiotensin converting enzyme activity (mediates angiotensin‐II and bradykinin production and vasoconstriction), change early in the course of illness (Casey et al. 1981), have some predictive value for the development of acute lung injury (Rubin et al. 1990; Bhandari et al. 2006), and correlate with prognosis (Table 1) (Ware et al. 2004; Calfee et al. 2012). These upstream signals promote leukocyte and platelet adhesion, amplify local inflammation, and induce endothelial cell structural changes resulting in intercellular gaps and increased vascular permeability (Matthay et al. 2019; Dudek and Garcia 2001; Schnells et al. 1980). Pulmonary vascular occlusion (Tomashefski et al. 1983), which can be present in arteries, capillaries, and veins, results from platelet and leukocyte plugs and likely increases pulmonary vascular resistance (PVR) (Ryan et al. 2014; Looney et al. 2006; Powe et al. 1982; Zapol et al. 1977). In situ thrombosis and anti‐fibrinolysis are further evidenced by increased levels of plasminogen activator inhibitor‐1 (PAI‐1), and reduced protein C, both found on the endothelial cell surface (Table 1). These changes in PAI‐1 and protein C are associated with clinical outcomes in ARDS, and in a sheep model of acute lung injury, administration of activated protein C improved lung edema and pulmonary artery pressures (Tsangaris et al. 2009; Matthay and Ware 2004; Waerhaug et al. 2008). Autopsy studies from patients with severe SARS‐CoV‐2 pneumonia (COVID‐19) and ARDS showed extensive capillary thromboses, more pronounced than in specimens from patients with influenza pneumonia (Ackermann et al. 2020).

**TABLE 1:** Selected biomarkers and mediators involved in vascular pathology in ARDS.

	Predominant source	Function
Inflammation		
IL‐6	Monocytes, macrophages, neutrophils, alveolar epithelium	Pro‐inflammatory cytokine (Matthay et al. 2019)
IL‐8	Monocytes, macrophages, neutrophils, alveolar epithelium	Chemokine (Matthay et al. 2019)
Soluble tumor necrosis factor receptor 1	Epithelium, macrophages	Reflects activation of TNF pathway activation (Matthay et al. 2019)
IL‐1β	Epithelium, macrophages, monocytes	Pro‐inflammatory cytokine (Matthay et al. 2019)
Vasoconstriction		
Nitric oxide	Endothelium, smooth muscle	Vasodilation (Price et al. 2012)
Endothelin‐1	Endothelium, epithelium, smooth muscle	Vasoconstriction (Price et al. 2012)
Prostanoids	Endothelium, epithelium, smooth muscle, platelets, leukocytes	Vasodilation/vasoconstriction (Price et al. 2012)
Vascular endothelial integrity		
Angiopoietin‐2	Endothelium, platelets	Promotes endothelial regression and vascular permeability (Fagiani and Christofori 2013)
Syndecan	Endothelial glycocalyx	Endothelial barrier protection and homeostasis (Gomez Toledo et al. 2025)
Leukocyte endothelial adhesion		
Intercellular adhesion molecule 1	Endothelium, epithelium, macrophages	Leukocyte recruitment (Gomez Toledo et al. 2025)
Vascular cell adhesion molecule 1	Endothelium	Leukocyte recruitment (Gomez Toledo et al. 2025)
E‐selectin	Endothelium	Leukocyte recruitment (Gomez Toledo et al. 2025)
P‐selectin	Endothelium	Leukocyte and platelet recruitment (Gomez Toledo et al. 2025)
Thrombosis and fibrinolysis		
Von Willebrand Factor	Endothelium, platelets	Platelet adhesion (Gomez Toledo et al. 2025)
Protein C	Liver	Anticoagulant (Price et al. 2012)
Plasminogen activator inhibitor‐1	Endothelium, macrophages	Antifibrinolytic (Price et al. 2012)

**FIGURE 3:**
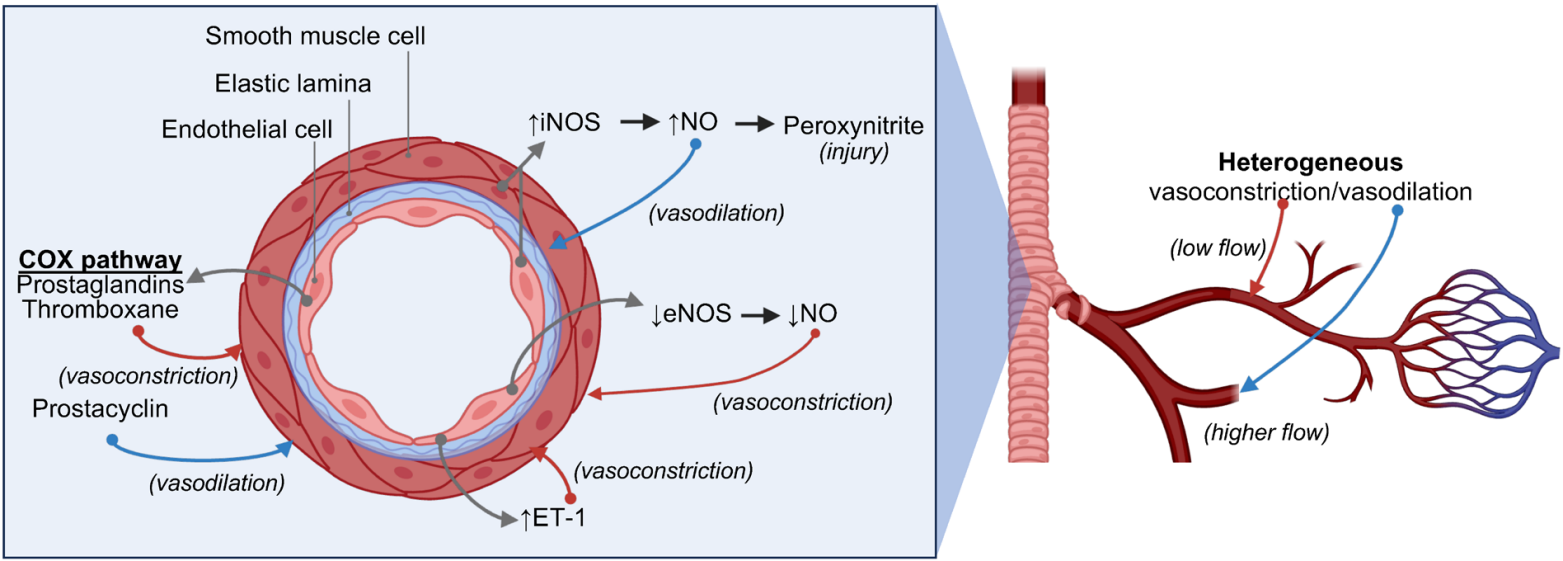
Mediators of heterogeneous vascular function. Nitric oxide (NO) is produced by endothelial and smooth muscle cells to promote vasodilation (blue arrows), though differential expression or induction of NO synthase across vascular beds can lead to heterogeneous areas of vasodilation or vasoconstriction (red arrows). Excessive NO leads to production of toxic reactive nitrogen species like peroxynitrite. Prostanoids and Endothelin‐1 (ET‐1), produced by endothelial cells, mediate vasoreactivity. COX, cyclooxygenase; eNOS, endothelial nitric oxide synthase; iNOS, inducible nitric oxide synthase.

An imbalance in vasoactive mediators like nitric oxide (NO), endothelin‐1 (ET‐1), and the COX pathway plays a significant role (Figure 3, Table 1) (Ryan et al. 2014). NO is produced by endothelial and smooth muscle cells, acting through soluble guanylate cyclase to promote vasodilation (Price et al. 2012). In ARDS, NO production can be heterogeneous. Decreased expression of endothelial NO synthase and less NO production result in areas of vasoconstriction (Albertine et al. 1999), while increased inducible NO synthase (Albertine et al. 1999; Griffiths et al. 1995) leads to the development of peroxynitrite and further injury (Freeman 1994). ET‐1 is produced by endothelial and smooth muscle cells and is a potent vasoconstrictor (Ryan et al. 2014). ET‐1 levels are markedly elevated in ARDS and decrease as pulmonary arterial pressures improve (Langleben et al. 1993). In a porcine model of acute lung injury, ET‐1 and PVR rise following meconium aspiration, while ET‐1 blockade mitigates pulmonary vasoconstriction (Shekerdemian et al. 2004). The COX pathway metabolizes arachidonic acid into prostaglandins, prostacyclin, and thromboxane, all of which affect vessel tone. In a sheep model, intravenous endotoxin infusion led to increased thromboxane‐A2 levels and an acute rise of pulmonary artery pressures (Hüttemeier et al. 1982).

**FIGURE 4:**
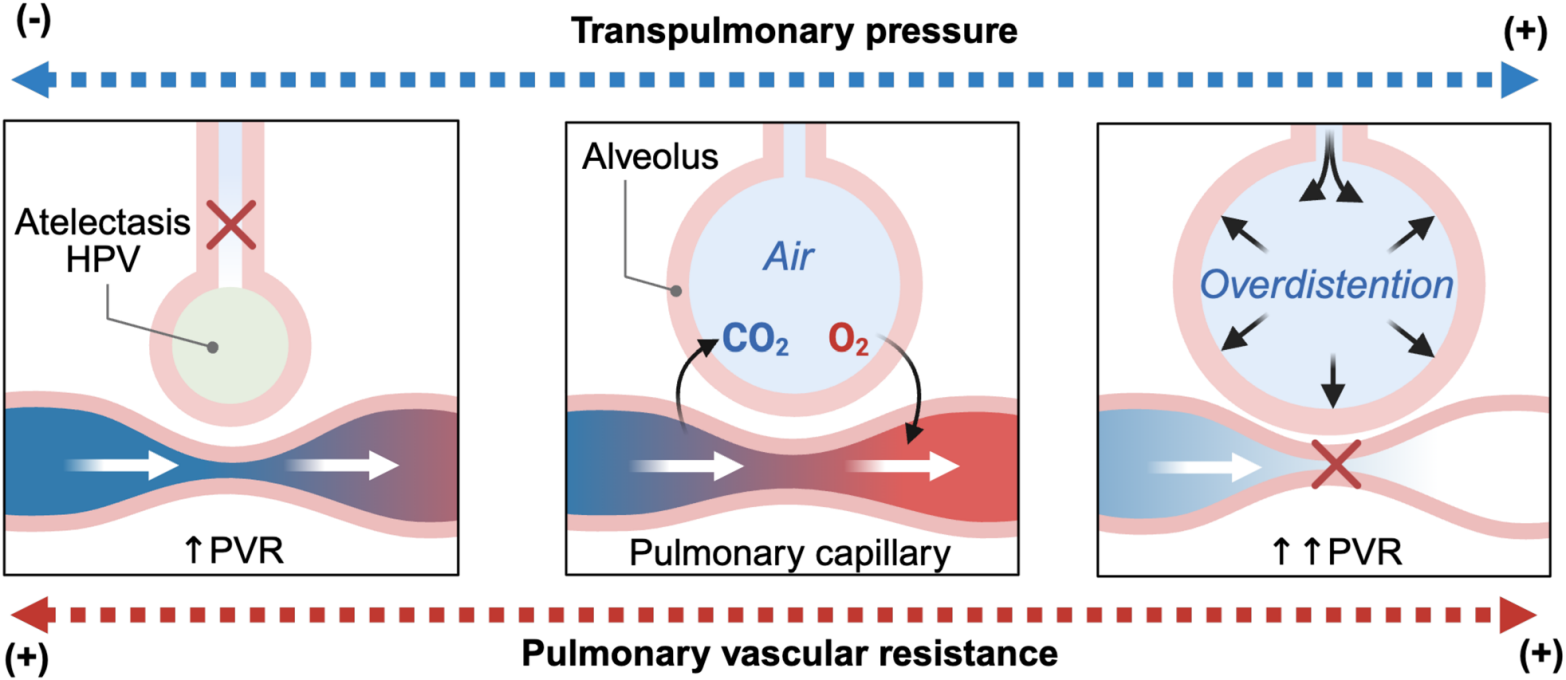
Positive pressure ventilation has a U‐shaped relationship with pulmonary vascular resistance. During low lung volumes and atelectasis (negative transpulmonary pressure), hypoxic pulmonary vasoconstriction (HPV) leads to a high pulmonary vascular resistance (PVR), while high volumes and alveolar overdistention (high positive transpulmonary pressure) lead to compression of alveolar capillaries and high PVR.

Hypoxic pulmonary vasoconstriction plays a complex role in the elevation of PVR (Figure 4). Hypoxia leads to Ca‐mediated Ca‐release and constriction of pulmonary vascular smooth muscle (Ryan et al. 2014; Moudgil et al. 2005). This is a protective mechanism which shunts blood towards well aerated areas of the lung, maintaining a near normal ventilation: perfusion ratio, but it contributes to increased PVR (Marshall et al. 1994). In ARDS, experiments show a significant increase in pulmonary pressures and PVR in acute hypoxia, partially mitigated by increased fraction of oxygen delivered through the ventilator (Bachofen and Weibel 1982) or by extracorporeal membrane oxygenation (Marshall et al. 1994; Benzing et al. 1997).

##### 1.1.1.2 Clinical Manifestations

Through the pathways described above, pulmonary vascular flow disturbance in ARDS is commonplace. Indeed, early reports found elevated PVR in every patient with severe ARDS (Zapol and Snider 1977). Myriad studies since, using pulmonary artery catheterization or echocardiography, document pulmonary hemodynamic changes in ARDS. In the era before lung protective ventilation, at least mild–moderate elevations in pulmonary artery pressures were seen in most patients (Villar et al. 1989; Suchyta et al. 1992; Squara et al. 1998; Osman et al. 2009). Even after lung protective ventilation became the standard of care, elevated pulmonary artery pressures could be present in > 90% of patients with severe ARDS (Beiderlinden et al. 2006). Analyzing data from the fluid and catheter trial (FACTT) of ARDS, investigators found > 70% of patients had an elevated transpulmonary gradient (Bull et al. 2010). Importantly, though the association between pulmonary artery pressures and mortality in ARDS is evident in some studies and not others (Ryan et al. 2014), indices of increased PVR are clearly associated with mortality, and reflect the vascular pathology in ARDS (Bull et al. 2010).

Pulmonary vascular hemodynamics reflect the degree of pulmonary vascular damage in ARDS. Increased dead space fraction (reflecting the proportion of tidal breath that does not interact with the alveolar‐capillary membrane to participate in CO_2_ exchange) resulting from injury of pulmonary capillaries is associated with mortality in ARDS (Nuckton et al. 2002; Sinha et al. 2019). Additionally, dead space fraction is correlated with pulmonary artery pressures (Cepkova et al. 2007) and PVR (Papolos et al. 2019). The earliest analyses of pulmonary artery catheter data in ARDS reported that PVR tended to fall in survivors, compared to non‐survivors (Zapol and Snider 1977). In a longitudinal analysis of hemodynamic data of the FACTT trial, survivors showed normalizing pulmonary artery compliance compared to non‐survivors (Metkus et al. 2017). Therefore, evidence of pulmonary vascular dysfunction may be a useful metric for tracking disease severity and potentially response to therapy in ARDS (Ryan et al. 2014). Echocardiographic methods for measuring pulmonary vascular resistance have been well described (Abbas et al. 2003; Lindqvist et al. 2011).

##### 1.1.1.3 Management

As the above pathophysiology has become clear, multiple therapies targeting the pulmonary vasculature have been tested in small clinical trials, with few being randomized. Though agents are uniformly associated with improved pulmonary vascular hemodynamics, their toxicity, off‐target effects, or hemodynamic instability have rendered all of them counterproductive or without clinical outcome benefits (Table 2) (Price et al. 2012). The effects of positive pressure ventilation on alveolar recruitment/distention, pulmonary vascular, and right ventricular (RV) function will be discussed in a separate section.

**TABLE 2:** Summary of studies evaluating targeted cardiac, pulmonary and systemic vascular therapies in ARDS.

Therapy	Mechanism	Disease	Subject	Study design	Outcome/result	References
*Anatomical near field*						
Pulmonary vasculature						
BQ‐123	Endothelin‐1 receptor antagonist, vasodilation	ARDS	Pig	Experimental animal study	↓ PVR	(Langleben et al. 1993)
Oxygen						
Fraction of inspired O_2_	Relief of HPV	ARDS	Human	Prospective non‐randomized	↓ PVR	(Marshall et al. 1994)
Fraction of delivered O_2_ by ECMO	Relief of HPV	ARDS	Human	Prospective non‐randomized	↓ PVR	(Cepkova et al. 2007)
Sildenafil	Phosphodiesterase‐5 inhibitor, vasodilation	ARDS	Human	Prospective non‐randomized	↓ PA pressures, no difference in oxygenation, ↑ hypotension	(Cornet et al. 2010)
Prostacyclin	Intravenous prostacyclin receptor agonist, vasodilation	ARDS	Human	Prospective non‐randomized	↓ PA pressures, no difference in oxygenation, ↑ hypotension	(Cornet et al. 2010)
Inhaled nitric oxide	Nitric oxide, vasodilation	ARDS	Human	RCT, comparator: Placebo	↓ PVR, ↑ RV function, ↑ cardiac output, No difference in mortality	(Bhorade et al. 1999; Dellinger et al. 1998; Taylor et al. 2004)
Inhaled prostaglandin E1	E‐prostanoid receptor agonost, vasodilation	ALI	Human	Prospective non‐randomized	↑ systemic oxygenation, No difference PA pressures	(Meyer et al. 1998)
Iloprost	Inhaled prostacyclin receptor agonist, vasodilation	ARDS	Human	RCT, comparator: Placebo	↑ systemic oxygenation, no difference in mortality	(Haeberle et al. 2023)
Coagulation/thrombosis						
Activated Protein C	Anticoagulation	ALI	Human	RCT, comparator: Placebo	No difference in clinical outcomes, no difference in mortality	(Liu et al. 2008)
Nebulized heparin	Anticoagulation	With or at risk for ARDS	Human	RCT, comparator: Placebo	Better lung injury score, no difference in mortality	(Dixon et al. 2021)
ALT‐836	Tissue factor antagonist, anticoagulation	Sepsis induced ALI or ARDS	Human	Phase I RCT, comparator: Placebo	Tolerable	(Morris et al. 2012)
Thrombomodulin	Anticoagulation	ARDS in children	Human	Feasibility	Feasible	(Hirata et al. 2021)
*Right ventricle*						
Mechanical ventilation						
PEEP	To optimized lung compliance	ARDS	Human	Prospective non‐randomized	↑ RV function	(Schmitt et al. 2001)
PEEP	To optimized transpulmonary pressure	ARDS	Human	Prospective non‐randomized	↑ RV function	(Vedrenne‐Cloquet et al. 2024)
Prone positioning	Optimized alveolar ventilation/recruitment	ARDS	Human	Prospective non‐randomized	↓ PVR, ↑ cardiac index	(Jozwiak et al. 2013)
Dobutamine	Inotrope, peripheral vasodilator	ARDS, septic shock	Human	Prospective non‐randomized	Improved extravascular lung water, decreased vasopressor dose	(Zhou et al. 2016)
Levosimendan	Inotrope, peripheral and pulmonary vasodilator	ARDS, septic shock	Human	RCT, comparator: Placebo	↓ PVR, ↑ cardiac index, ↑ RV function	(Morelli et al. 2006)
*Anatomical far field*						
Resistance arterioles						
NG‐methyl‐L‐arginine‐Hydrochloride	Non‐selective NOS inhibition	Septic shock	Human	RCT, comparator: Placebo	↑ SVR, ↑ blood pressure	(Bakker et al. 2004)
Methylene blue	NO scavenging, eNOS inhibition	Septic shock	Human	Prospective non‐randomized	↑ SVR, ↑ blood pressure	(Daemen‐Gubbels et al. 1995)
Methylene blue	NO scavenging, eNOS inhibition	Septic shock	Human	RCT, comparator: Placebo	↑ SVR, ↓ vasopressor dose, ↓ ICU stay, no difference in mortality	(Ibarra‐Estrada et al. 2023)
Methylene blue	NO scavenging, eNOS inhibition	Septic shock	Human	Enrolling RCT, comparator: Placebo	NA	(Luo et al. 2026)
High dose hydroxycobalamin	NO scavenging, eNOS inhibition	Septic shock	Pig	Experimental animal study	Improved vasoplegia, blood pressure and SVR	(Bebarta et al. 2019)
High dose hydroxycobalamin	NO scavenging, eNOS inhibition	Septic shock	Human	Phase II RCT, comparator: Placebo	↓ vasopressor dose, no difference in vasopressor‐free days	(Patel et al. 2023)
Terminal arterioles						
Normal saline or albumin	Intravascular volume expansion	Severe sepsis	Human	Prospective non‐randomized	Improved sublingual microvascular function	(Ospina‐Tascon et al. 2010)
Hydroxyethyl starch	Intravascular volume expansion	Severe sepsis	Human	RCT, comparator: Normal saline	Improved sublingual microvascular function	(Dubin et al. 2010)
Drotecogin alpha	Activated Protein C, anticoagulation	Severe sepsis	Human	Prospective non‐randomized	Improved sublingual microvascular function	(De Backer et al. 2006)
Drotecogin alpha	Activated Protein C, anticoagulation	Severe sepsis	Human	RCT, comparator: Placebo	No difference in mortality	(Ranieri et al. 2012)
Dobutamine	Inotrope, peripheral vasodilator	Septic shock	Human	RCT, comparator: Placebo	No difference in microvascular function	(Hernandez et al. 2013)
Levosimendan	Inotrope, peripheral vasodilator	Septic shock	Human	RCT, comparator: Dobutamine	Improved microvascular function	(Hernandez et al. 2013)
Intravenous Iloprost	Intravenous prostacyclin receptor agonist, vasodilation	Septic shock, endotheliopathy	Human	RCT, comparator: Placebo	No difference in organ dysfunction, no difference in microvascular function, trend towards more adverse effects	(Bestle et al. 2024)
Intravenous Iloprost	Intravenous prostacyclin receptor agonist, vasodilation	Septic shock, microvascular dysfunction	Human	RCT, comparator: Placebo	No difference in organ dysfunction, no difference in microvascular function, trend towards more adverse effects	(Legrand et al. 2025)
Targeted microvascular hemodynamics	Target: inotropes, inodilators, steroids	Septic shock	Human	RCT, comparator: Macrovascular targets (mean arterial pressure, cardiac index, central venous O_2_)	No difference in organ failure resolution	(van der Voort et al. 2015)
Personalized resuscitation	Targeted normalization of capillary refill	Septic shock	Human	RCT, comparator: Normalization of lactate	High likelihood of mortality benefit	(Zampieri et al. 2020; Hernández et al. 2019)
Personalized resuscitation	Targeted normalization of capillary refill	Septic shock	Human	RCT, comparator: Usual care	No difference in mortality, ↓ duration of vital support	(ANDROMEDA‐SHOCK‐2 Investigators for the ANDROMEDA Research Network, Spanish Society of Anesthesiology, Reanimation and Pain Therapy (SEDAR), and Latin American Intensive Care Network (LIVEN), et al. 2025)

Abbreviations: ALI, acute lung injury; ICU, intensive care unit; PA, pulmonary artery; PVR, pulmonary vascular resistance; RCT, randomized clinical trial; RV, right ventricle; SVR, systemic vascular resistance.

Counteracting pulmonary vasoconstriction with vasodilators has been met with mixed results. Systemic administration of sildenafil, a phosphodiesterase‐5 inhibitor, or prostacyclin results in lower pulmonary artery pressures and PVR at the expense of worsened oxygen shunt fraction (likely through blunting of hypoxic pulmonary vasoconstriction) and significant systemic hypotension, in small non‐randomized trials of ARDS (Cornet et al. 2010; Radermacher et al. 1990). Inhaled administration of pulmonary vasodilators minimizes systemic bioavailability and side effects while targeting pulmonary vasculature directly. Inhaled NO reduces PVR and improves RV function and cardiac output in ARDS (Bigatello et al. 1994; Fierobe et al. 1995; Bhorade et al. 1999). For example, in a non‐randomized trial of 26 patients with RV failure (12 of whom had ARDS), inhaled NO improved PVR, cardiac output, and hypoxia (Bhorade et al. 1999). However, no randomized placebo‐controlled trial of inhaled NO in ARDS has shown a clear benefit in mortality or patient‐centered outcomes, even when patients with pulmonary organ failure alone are selected (Dellinger et al. 1998; Taylor et al. 2004). Inhaled prostaglandin E1 and iloprost (a synthetic prostacyclin) are both associated with improved systemic oxygenation (Meyer et al. 1998; Siddiqui et al. 2013). A recent randomized placebo‐controlled trial of severe COVID and non‐COVID ARDS treated with inhaled iloprost found no improvement in clinical outcomes compared to placebo (Haeberle et al. 2023).

Regarding targeting of intravascular coagulation and in situ thrombosis, few trials exist. A randomized placebo‐controlled trial of activated protein C in 75 patients with acute lung injury found improved dead space fraction with therapy but not evidence of improved clinical outcomes (Liu et al. 2008). A recent placebo‐controlled trial of nebulized heparin in 256 mechanically ventilated patients with, or at risk for, ARDS found improvement in lung injury score with therapy, but no impact on mortality (Dixon et al. 2021). Trials to date evaluating therapies modulating tissue factor (Morris et al. 2012) and thrombomodullin (Hirata et al. 2021) are limited to feasibility and safety endpoints.

#### 1.1.2 Right Ventricle

##### 1.1.2.1 Pathophysiology

Through pulmonary vascular involvement, RV function is central to ARDS cardiovascular pathophysiology. The RV has evolved to receive blood from a low‐pressure venous system (preload) and pump it into a low impedance, distensible pulmonary vasculature (afterload) (Haddad, Hunt, et al. 2008). As a result, the RV is thin‐walled and compliant compared to the left ventricle (LV), and its primary adaptive response to an acute increase in afterload is dilatation (Haddad, Doyle, et al. 2008). The acute development of pulmonary vascular dysfunction during ARDS pathologically increases RV afterload. If the stress on the RV continues or worsens, inexorable RV dilation leads to a left‐sided interventricular septal shift, an impairment of LV filling and output which leads to impaired RV coronary flow, increased RV afterload, and an ischemic vicious cycle (Konstam et al. 2018).

Outside of the biology implicating the pulmonary vasculature as above, limited evidence suggests that systemic inflammation may additionally mediate cardiac dysfunction in ARDS. In a mouse model of acute lung injury through lipopolysaccharide (LPS) inhalation, wild type mice exhibited significant reductions in cardiac output, while IL (interleukin)‐6 knock‐out mice did not, and exogenous IL‐6 recapitulated the reduction in cardiac output (Suda et al. 2011). In a mouse model of COVID‐ARDS, both infection with SARS‐CoV‐2 and a virus‐free trigger led to worsened cardiac output and LV ejection fraction along with specific expansion of cardiac macrophage subsets, which were also elevated in human cardiac autopsies from COVID‐19 (Grune et al. 2024). Furthermore, treating mice with an anti‐tumor necrosis factor alpha (TNF‐alpha) antibody mitigated macrophage expansion and cardiac dysfunction. Neither of these studies specifically measured RV function.

Since sepsis frequently coexists with or triggers ARDS, the biology thought to underpin septic cardiomyopathy, which may present as RV dysfunction in 48% of patients, is pertinent (Zakynthinos et al. 2025; Sato et al. 2025; Lanspa et al. 2021). In sepsis, triggered by damage‐associated molecular patterns (DAMPs) and pathogen‐associated molecular patterns (PAMPs), an exuberant activation of the NF‐κB pathway results in transcription of pro‐inflammatory cytokines like IL‐1β, IL‐6, and TNF‐alpha (Table 1) (Razazi et al. 2019). Downstream effects like excessive NO production (Ungureanu‐Longrois, Balligand, Kelly, and Smith 1995; Ungureanu‐Longrois, Balligand, Simmons, et al. 1995), mitochondrial dysfunction (Xu et al. 2012), disrupted calcium handling (Martin et al. 2016) and myocardial edema (Muehlberg et al. 2022) have been implicated in septic cardiomyopathy.

##### 1.1.2.2 Clinical Manifestations

The discovery of ventilator‐induced lung injury (VILI) and how limiting tidal volume and alveolar over‐distention improves clinical outcomes led to a seismic shift in mechanical ventilator management in ARDS (Slutsky and Ranieri 2013). This change in practice is suspected to have played a significant role in curbing the incidence of its most severe form of RV dysfunction—acute cor pulmonale (Jardin and Vieillard‐Baron 2007). Despite this, RV dysfunction overall remains prevalent (Zochios et al. 2017; Boissier et al. 2013). RV failure is defined as a clinical syndrome that results from the inability of the RV to fill or eject blood (RV dysfunction), with the cardinal features of elevated right atrial pressure, fluid retention and decreased output (Haddad, Doyle, et al. 2008). Several echocardiographic parameters define RV dysfunction, such as the tricuspid annular plane systolic excursion, RV fractional area change and RV free wall strain. It bears emphasizing that the RV is crescent‐shaped and incompletely imaged in any single echocardiographic view (Mukherjee et al. 2025). Its motion is complex, and function depends on coordinated contraction of separate longitudinal, radial and anteroposterior axes (Haddad, Hunt, et al. 2008). Therefore, commonly used parameters can, at best, provide an incomplete assessment of function—better approximated only on three‐dimensional imaging (Mukherjee et al. 2025; O'Donnell et al. 2023). Depending on the combination of parameters used, RV dysfunction may be as prevalent as 55% in contemporary cohorts of ARDS (Zochios et al. 2017).

Cor‐pulmonale is defined on echocardiography as the presence of RV dilatation in association with dyskinesia of the interventricular septum as a response to increased RV afterload (Vieillard‐Baron et al. 2001; Boissier et al. 2013). In a study of moderate–severe ARDS, acute cor‐pulmonale was associated with receipt of prone ventilation and inhaled NO and independently associated with mortality even when tidal volume and pressure were limited (Boissier et al. 2013). However, in other analyses focused on populations with limited alveolar distention, the association between acute cor‐pulmonale and mortality was attenuated (Vieillard‐Baron et al. 2001). The reason for these conflicting results is not clear, but it is possible that the presence of marked echocardiographic abnormalities in acute cor‐pulmonale led to significant changes in care that influenced mortality (Repessé et al. 2015). Notably, specific echocardiographic parameters of RV dysfunction are associated with mortality, which underscores its continued importance (Shah et al. 2016). Hemodynamic definitions of RV dysfunction from pulmonary artery catheterization have similarly been inconsistently associated with mortality. An elevated right atrial pressure above pulmonary capillary wedge pressure (a surrogate for left atrial pressure) was independently associated with in‐hospital mortality in two single center studies (Monchi et al. 1998; Osman et al. 2009). However, secondary analyses of FACTT found weak or no associations between RV function and mortality (Bull et al. 2010; Hockstein et al. 2024). Therefore, RV dysfunction is an important complication of ARDS; it is associated with poor clinical outcomes, but its definition and clinical context are important confounders.

##### 1.1.2.3 Considerations During Mechanical Ventilation

Spontaneous respiration is generally associated with salutary effects on RV preload, RV afterload and performance (Alviar et al. 2018). Positive pressure ventilation exerts a reversal of the usual respiro‐hemodynamic swings. Though in a healthy state, without exuberant alveolar distention, positive pressure ventilation exerts minimal detrimental effects; during ARDS this therapy can either decompensate or ameliorate RV function (Repessé et al. 2015; Vieillard‐Baron et al. 2013). Positive pressure has separate and discordant effects on RV and LV afterload. It decreases LV afterload by differentially increasing intrathoracic pressure compared to intraabdominal pressure and therefore improving a forward pressure gradient from the LV towards the peripheral vasculature. It can increase RV afterload in the presence of alveolar over‐ or under‐ distention. The alveoli reside under competing tensions towards collapse and expansion. In ARDS, lung injury results in accumulation of protein‐rich alveolar fluid, surfactant dysfunction, and reduced lung compliance which, along with patient positioning, lead to regional atelectasis, consolidation, reduced functional residual capacity and hypoxia (Matthay et al. 2019). This hypoxia results in increased PVR. Positive pressure ventilation can mitigate this effect by alleviating atelectasis or can exacerbate it through excessive regional alveolar over‐distention, which can then lead to capillary collapse, and again a rise in PVR (Figure 4) (West and Luks 2015). Slobod et al. (2026) provide an excellent, more extensive review of right ventricular hemodynamics in ARDS.

The U‐shaped relationship between lung volume and PVR means a nuanced ventilation strategy is paramount to optimizing cardiopulmonary function in ARDS (Suter et al. 1975). This is illustrated by a growing body of experimental and clinical evidence on positive end‐expiratory pressure (PEEP) titration, lung recruitment maneuvers, and prone positioning.

Experimental animal studies suggest a PEEP that promotes atelectasis is associated with worse pulmonary vascular dysfunction and mortality. In a porcine model of ARDS, 36 pigs were randomized 1:1:1 to a PEEP titrated to low overdistention (biasing towards atelectasis), low collapse (biasing towards overdistention) and a PEEP in between (Sousa et al. 2024). Compared to the other arms, the low overdistention group had markedly higher mortality, PVR, and vasopressor requirements (Sousa et al. 2024). Clinical data support a similarly individualized approach. In a prospective study of patients with ARDS and pulmonary artery catheters, esophageal manometry, and echocardiography, investigators systematically increased PEEP while evaluating PVR, right and left ventricular function, and lung recruitment (Cappio Borlino et al. 2024). They found that among patients with unchanged or high lung recruitability, PVR was unchanged with every increase in PEEP. However, in low lung recruitability, increases in PEEP were met with increased PVR and RV dilatation (Cappio Borlino et al. 2024). A separate prospective study showed that increasing PEEP to target the highest lung compliance improved RV stroke index compared to lower or higher PEEP (Schmitt et al. 2001). Plateau pressures below 27cmH_2_O were associated with lower incidence of acute cor pulmonale and mortality compared to higher pressures (Jardin and Vieillard‐Baron 2007). Similarly, a prospective study of 46 adults and children with ARDS using esophageal manometry to estimate transpulmonary pressure found that higher transpulmonary pressure (an index of alveolar overdistension) was associated with greater odds of RV dysfunction (Vedrenne‐Cloquet et al. 2024).

The effect of lung recruitment maneuvers and prone positioning on RV performance has also been evaluated prospectively. In a study of 34 patients with moderate–severe ARDS, RV size and function were evaluated before and after a lung recruitment maneuver (Lambour et al. 2025). Investigators found that respiratory system compliance increased, RV size decreased, and RV function improved after recruitment. However, among non‐responders, such maneuvers can lead to hemodynamic instability (Mercado et al. 2018). Lastly, in a prospective study of 18 patients with ARDS, prone positioning was associated with improved oxygenation as well as PVR and cardiac index (Jozwiak et al. 2013).

##### 1.1.2.4 Management

Outside of the clinical trials discussed in the pulmonary vasculature section above, there have been no adequately powered randomized clinical trials of targeted therapies for RV dysfunction in ARDS evaluating patient‐centered clinical outcomes or mortality (Ganeriwal et al. 2023).

### 1.2 The Anatomical Far‐Field of the Lung: Systemic Cardio‐Vascular Interactions

The peripheral vascular arterial tree terminates in a highly branched structure with differential function across levels. Blood in the large arteries flows into numerous arterioles, highly muscular 10–15 μm vessels providing the primary level of resistance to blood flow (vasomotor tone) (Hall and Guyton 2025). Blood then flows into further branching terminal arterioles measuring 5–9 μm, where surrounding smooth muscle is present at intermittent points (i.e., regional vasoconstriction can affect flow heterogeneously), and ultimately into muscle‐free capillaries (where gas and nutrient exchange predominantly occurs) (Hall and Guyton 2025). Generally, the peripheral vasculature is composed of: The glycocalyx facing the lumen (made up of glycosaminoglycans that protect the endothelial cells and support homeostasis) (Tarbell and Cancel 2016), a single layer of endothelial cells, vascular smooth muscle (present in arteries and arterioles) and the interstitium (containing collagen fibers, and proteoglycan filaments, providing intercellular structural integrity) (Hall and Guyton 2025; Miranda et al. 2016).

Analogous to endothelial pathology evident in the lung, systemic vascular permeability frequently increases in ARDS and contributes to multisystem organ failure (Matthay et al. 2019). Plasma angiopoietin‐2, a marker of endothelial injury elevated in direct and indirect (sepsis‐induced) ARDS, is significantly higher in indirect ARDS, though its prognostic value is maintained in both (Calfee et al. 2015). This finding suggests that the source of lung injury affects the extent of systemic endothelial involvement. While these mechanisms have been characterized predominantly in sepsis and septic shock, their relevance to ARDS is suggested by molecular subphenotypes of ARDS, distinguished in part by differential prevalence of shock and plasma markers of vascular instability, being conserved in sepsis (Sinha et al. 2023). Using sepsis as a model, systemic vascular function in ARDS is affected at the level of resistance arterioles, terminal arterioles and capillaries, and interstitium, manifesting as shock, micro‐circulatory dysfunction, and edema. Notably, non‐septic models show that lung injury alone can lead to systemic vascular dysfunction, independent of sepsis. Akin to pathology in the pulmonary vasculature in ARDS, heterogeneous vasodilation and vasoconstriction, thrombosis, and loss of capillary density are central to the systemic vascular dysfunction (Miranda et al. 2016).

#### 1.2.1 Resistance Arterioles

##### 1.2.1.1 Pathophysiology

Systemic inflammation and endothelial injury generate a cascade of mediators that affect vascular smooth muscle tone (Figure 3). NO is generated largely by endothelial and inducible synthetases (eNOS and iNOS), which are present in endothelial and vascular smooth muscle cells, respectively (Table 1) (Lambden et al. 2018). Induction by local mediators (e.g., thrombin, bradykinin, and prostaglandins), systemic cytokines, and PAMPs leads to remarkable elevations in NO production (e.g., production by iNOS is increased 1000‐fold) (Levy et al. 2018). Indeed, non‐selective inhibition of NOS was associated with improved systemic vascular resistance (SVR) and blood pressure in a randomized controlled trial of septic shock (Bakker et al. 2004). Methylene blue and high‐dose IV hydroxycobalamin, acting through a combination of NO scavenging and inhibition of eNOS, led to improved SVR and blood pressure in sepsis (Daemen‐Gubbels et al. 1995; Kwok and Howes 2006; Bebarta et al. 2019). Prostacyclin is produced by endothelial cells, leads to platelet aggregation, and induces cAMP‐mediated vasodilation (Lambden et al. 2018). In vitro experiments show that PAMPs (like LPS) and inflammatory cytokines (IL‐1 and TNF‐alpha) promote generation of prostacyclin by the COX‐2 pathway in endothelial cells (Endo et al. 1988; Riedo et al. 1990). In a rat model of septic shock, treatment with selective prostacyclin receptor inhibition was associated with improved vasoplegia (Höcherl et al. 2008).

Stimulation of the sympathetic nervous system and hypothalamic–pituitary–adrenal axis in sepsis, in part by inflammatory cytokines, affects vasomotor tone (Levy et al. 2018). The initial response from these systems leads to release of catecholamines (norepinephrine and epinephrine), vasopressin, angiotensin‐II, and cortisol, necessary for maintenance of vascular responsiveness during shock. However, persistent stimulation can lead to receptor desensitization or decreased release of these molecules and vasoplegia. For example, in vitro exposure of canine hepatic endothelial cells to endotoxin reduced alpha‐adrenergic receptor sensitivity (Ghosh and Liu 1983). Angiotensin‐II receptor 1 was downregulated in a mouse model of sepsis, and inhibition of the NFkB pathway significantly decreased downregulation (Schmidt et al. 2010; Legrand et al. 2024). Interestingly, secondary analyses of the ATHOS‐3 trial of vasodilatory shock found that patients with ARDS had improved oxygenation, in addition to hemodynamics, when treated with angiotensin‐II compared to placebo suggesting it might augment hypoxic pulmonary vasoconstriction and improve ventilation: perfusion matching (Leisman et al. 2023). In patients with septic shock, serum vasopressin levels fell below normal levels and they had vascular hyper‐reactivity to vasopressin infusion (Landry et al. 1997). A mouse model of chronic sepsis revealed a decreased number of hepatic vasopressin receptors (Roth and Spitzer 1987). Finally, corticosteroid receptors potentiate response to catecholamines and angiotensin‐II (Prigent et al. 2004), and some evidence suggests that inflammatory cytokines affect the hypothalamic–pituitary–adrenal axis and cortisol production (Gaillard et al. 1990; Jäättelä et al. 1991).

##### 1.2.1.2 Clinical Manifestations

The above pathophysiology translates clinically into hypotension and shock (Fein et al. 1983). Across multiple trials of ARDS, the prevalence of shock or vasopressor requirement on enrollment is high, between 30% and 70% (National Heart, Lung, and Blood Institute Acute Respiratory Distress Syndrome (ARDS) Clinical Trials Network, et al. 2006; Siuba et al. 2024; Calfee et al. 2014; McAuley et al. 2014; National Heart, Lung, and Blood Institute PETAL Clinical Trials Network, et al. 2019). Although the prevalence of vasopressor use is associated with the source of lung injury, higher in sepsis‐related ARDS, direct ARDS has a prevalence of shock of 30% (Calfee et al. 2015). Hemodynamically, patients present with a low diastolic blood pressure, widened systemic pulse pressure, low SVR, and elevated cardiac output (Levy et al. 2018). In the FACTT trial of ARDS, in the context of 30% vasopressor use on enrollment, the mean cardiac index across arms was elevated ~4.2 L/min/m^2^, suggesting that the predominant etiology of shock was vasoplegic (National Heart, Lung, and Blood Institute Acute Respiratory Distress Syndrome (ARDS) Clinical Trials Network, et al. 2006; Metkus 2024). Prognosis in ARDS is aligned with multiorgan dysfunction and shock (Thille et al. 2013; Boyle et al. 2015). Interestingly, when viewed through the lens of molecular phenotyping, circulatory shock is the most common cause of death in hyperinflammatory compared to hypoinflammatory sepsis, where the attributable fraction of death from ARDS is higher (Evrard et al. 2024).

##### 1.2.1.3 Management

Guideline recommendations for management of vasoplegia during ARDS mirrors indications in septic shock (Prescott et al. 2026). Norepinephrine is recommended as first line vasopressor, followed by vasopressin, while a 3rd line agent should be guided by specific physiology encountered (e.g., inotropes for concomitant cardiogenic shock). Intravenous corticosteroids are recommended for persistent shock and high vasopressor requirements (Evans et al. 2021).

Therapies targeting NO or prostacyclin are associated with hemodynamic improvements but have no effect or detrimental effects on mortality (Table 2). A randomized controlled trial of ibuprofen (a non‐selective COX‐2 inhibitor) in patients with sepsis found reduced prostacyclin levels, but no effect on the development of shock or ARDS, nor mortality (Bernard et al. 1997). Non‐selective inhibition of NOS was associated with improved SVR and blood pressure in a randomized controlled trial of septic shock, though it was associated with increased mortality (López et al. 2004). A randomized clinical trial of methylene blue in 95 patients with septic shock found improved blood pressure, vasopressor dose, and shorter time to vasopressor discontinuation and ICU stay, without a difference in mortality (Ibarra‐Estrada et al. 2023). Recent meta‐analyses of vasodilatory shock, limited by a high proportion of observational data and heterogeneity in dosing and etiology of shock, have found consistent improvement in hemodynamics from methylene blue but inconsistent benefits in mortality (Arias‐Ortiz and Vincent 2024). A large trial is currently enrolling patients with high‐dose vasopressors in septic shock to compare methylene blue to a dextrose infusion (control) with the primary outcome of mortality (Luo et al. 2026). A phase‐II single‐center randomized controlled trial of intravenous hydroxycobalamin in 20 patients with septic shock found improved vasopressor requirements, but no difference in vasopressor‐free days (Patel et al. 2023).

#### 1.2.2 Terminal Arterioles and Capillaries

##### 1.2.2.1 Pathophysiology

Non‐septic experimental models of acute lung injury provide direct evidence that lung injury itself, independent of sepsis, leads to systemic vascular dysfunction mediated by inflammation and oxidative stress. In rodent models of VILI, high tidal volume ventilation decreases systemic vascular reactivity, including in isolated systemic arteries and the aorta, and increases mesenteric endothelial leukocyte migration, which are mitigated by superoxide scavengers (Aikawa et al. 2011; Martínez‐Caro et al. 2009; Nin et al. 2008). In a model of acute lung injury induced by hydrochloric acid aspiration, injured animals developed extensive ileal morphological alterations and elevated lymph‐to‐plasma protein ratios, indicating both remote organ injury and increased vascular permeability. Further, pre‐treatment with an anti‐leukocyte adherence antibody mitigated these changes (St John et al. 1993).

As in the pulmonary vasculature, endothelial cell activation and injury are central to the pathology observed (Figure 2) (Miranda et al. 2016). Toll‐like receptors on the endothelial surface interact with PAMPs and DAMPs after infection or tissue injury, which results in stimulation of inflammatory mediators through the NFkB pathway (Raia and Zafrani 2022). This response leads to the production of chemokines and cytokines, amplifying the inflammatory response and tissue recruitment of leukocytes (Table 1). Endothelial cell activation, cytokines, and local neutrophil production of reactive oxygen species lead to the shedding and damage of the glycocalyx, which exposes the endothelial surface (Raia and Zafrani 2022; Marechal et al. 2008). This damage exposes leukocyte adhesion molecules and releases vWF and tissue factor, further facilitating leukocyte recruitment and platelet aggregation (Miranda et al. 2016). This pro‐adhesive and pro‐thrombotic environment leads to microvascular thrombosis, capillary plugging, and impaired flow (Miranda et al. 2016; Secor et al. 2010). Additionally, glycocalyx degradation potentiates paracellular permeability and interstitial edema (Henry and Duling 2000; Chelazzi et al. 2015).

Dysregulated and heterogeneous production of NO alters microvascular blood flow and compounds endothelial injury. PAMPs lead to decreased production of NO through decreased expression and downregulation of eNOS (Lu et al. 1996; Zhou et al. 1997). Further, iNOS is differentially induced across tissue beds and organs. Areas where iNOS activity is high are more vasodilated and vice versa, contributing to arteriovenous shunting, tissue hypoperfusion, and hypoxia (Ince and Sinaasappel 1999). This robust production of NO increases the production of peroxynitrite, which both decreases NO bioavailability and produces a highly toxic molecule (Miranda et al. 2016). Heterogeneity in blood flow may be extreme, with neighboring areas of oxygenated vs. hypoxic tissues (Ellis et al. 2002; De Backer et al. 2002).

##### 1.2.2.2 Clinical Manifestations

Microvascular dysfunction is common in ARDS, associated with severity of hypoxia and with mortality, as it is in severe sepsis (Orbegozo Cortés et al. 2016; Chen et al. 2024; Ospina‐Tascón et al. 2020; De Backer et al. 2013). Interestingly, indices of systemic microvascular dysfunction (e.g., percentage of small vessels perfused and microcirculatory flow index) are highly associated with dead‐space fraction, suggesting both phenomena may reflect parallel vascular pathology in the periphery and the lungs, respectively (Ospina‐Tascón et al. 2020). Microvascular dysfunction correlates with organ dysfunction. In a prospective study of septic shock, persistent microvascular dysfunction in the sublingual circulation was correlated with organ dysfunction, even after resolution of shock, and was associated with mortality (Sakr et al. 2004). Clinically, this dysfunction may be evident by persistent hypoperfusion (e.g., elevated lactate or organ injury) despite optimized hemodynamics—loss of hemodynamic coherence (Ince 2015). Patients may present pale/cold skin or mottling (Ait‐Oufella et al. 2013), and prolonged capillary refill time (Huang et al. 2023). Direct clinical assessment of microvascular dysfunction is limited by a lack of available point‐of‐care serum biomarkers (aside from lactate), and need for specialized and sometimes cumbersome imaging techniques (Miranda et al. 2016). Longitudinal perfusion assessments in septic shock patients revealed that microvascular dysfunction is slowest to normalize, far longer than lactate (Hernandez et al. 2014). Endothelial markers of leukocyte adhesion (Table 1) such as intercellular adhesion molecule‐1 (ICAM‐1), vascular cell adhesion molecule‐1 (VCAM‐1), E‐selectin and P‐selectin are all associated with presence of sepsis, though association with mortality is modest (Zonneveld et al. 2014). Elevated syndecan‐1 (a component of the glycocalyx, Table 1) was associated with organ failure, vasopressor intensity, development of ARDS and mortality in a large cohort of patients with severe sepsis (Murphy et al. 2017). Another glycosaminoglycan, chondroitin sulfate, is associated with both mortality and heterogeneity in treatment effect of a restrictive vs. liberal resuscitation strategy in sepsis (Oshima et al. 2026).

##### 1.2.2.3 Management

There have been no randomized clinical trials targeting systemic microvascular dysfunction directly in ARDS patients. Sepsis and septic shock are replete in feasibility and biologic studies, but randomized clinical trials evaluating clinical outcomes, including mortality, are scant. Ultimately, no specific agents targeting microvascular dysfunction have been associated with improved mortality in sepsis; however, personalized strategies targeting capillary refill time are associated with benefit (Table 2) (Zampieri et al. 2020; ANDROMEDA‐SHOCK‐2 Investigators for the ANDROMEDA Research Network, Spanish Society of Anesthesiology, Reanimation and Pain Therapy (SEDAR), and Latin American Intensive Care Network (LIVEN), et al. 2025).

Interventions aimed at improving indices of microvascular function have generally been successful in human studies. Intravascular volume expansion through normal saline (or albumin) (Ospina‐Tascon et al. 2010), or hydroxyethyl starch (Dubin et al. 2010) was associated with improvements in sublingual microvascular function in patients with severe sepsis. Targeting intravascular thrombosis through activated protein C was associated with improvement in sublingual microvascular function in a prospective non‐controlled study of sepsis patients (De Backer et al. 2006); however, a large randomized controlled trial in septic shock found no improvement in mortality compared to placebo, even among patients with severe protein C deficiency at baseline (Ranieri et al. 2012). Clinical trials of inodilators in septic shock have had mixed results, with dobutamine having no effect on microvascular function compared to placebo (Hernandez et al. 2013), while levosimendan had modest improvements in microvascular function compared to dobutamine (Hernandez et al. 2013). NOS stimulation through iloprost was tested in two randomized clinical trials of septic shock and severe endotheliopathy (Bestle et al. 2024) or microvascular dysfunction (Legrand et al. 2025) evaluating change in organ function, and neither found an improvement (Bestle et al. 2024; Legrand et al. 2025). Notably, both trials showed a trend towards more serious adverse events in the intervention arm. Regarding comprehensive resuscitation strategies in sepsis, results are also mixed. A randomized controlled trial of 99 patients with septic shock, comparing resuscitation targeting macrovascular hemodynamics (mean arterial pressure, cardiac index, and central venous O_2_ saturation) vs. microcirculatory function using inotropes/inodilators and steroids did not find a difference in organ failure resolution (van der Voort et al. 2015). The ANDROMEDA‐SHOCK trial randomized patients with septic shock to a personalized strategy targeting normalization of capillary refill time vs. lactate and found a numerically lower incidence of death in the primary analysis, while a secondary Bayesian analysis found a high likelihood of benefit for the personalized strategy (Zampieri et al. 2020; Hernández et al. 2019). In this context, the ANDROMEDA‐SHOCK 2 trial again tested a personalized strategy targeting normalized capillary refill time against usual care and found the intervention arm was associated with similar mortality but lower duration of vital support (ANDROMEDA‐SHOCK‐2 Investigators for the ANDROMEDA Research Network, Spanish Society of Anesthesiology, Reanimation and Pain Therapy (SEDAR), and Latin American Intensive Care Network (LIVEN), et al. 2025).

### 1.3 Bidirectional Interactions Between Lung‐Heart‐Systemic Vascular Derangements

Though there is ample mechanistic and clinical evidence linking lung injury in ARDS to the development of right and left cardiac injury and dysfunction, evidence of whether right or left‐sided cardiac dysfunction can in turn compound lung injury is much sparser and confounded. Nonetheless, this bidirectional relationship has face validity. RV dysfunction resulting from the above mechanisms may lead to venous congestion, decreased right‐sided cardiac output, or both, resulting in organ congestion and hypoperfusion. Separately, pre‐morbid or new LV dysfunction, from septic cardiomyopathy, acute myocardial ischemia, or ventricular interdependence during concomitant RV dysfunction, can increase left‐sided filling pressures and worsen left‐sided cardiac output. At least three mechanisms by which cardiovascular dysfunction could then worsen lung injury stand out. First, in the context of the endothelial permeability that defines ARDS, concomitantly elevated left‐sided filling pressures could further impair lung function and prolong respiratory failure; indeed, over 30% of patients enrolled in FACTT had elevated pulmonary capillary wedge pressure, suggesting hydrostatic and permeability mechanisms of pulmonary edema often coexist (National Heart, Lung, and Blood Institute Acute Respiratory Distress Syndrome (ARDS) Clinical Trials Network, et al. 2006). Second, akin to non‐pulmonary sepsis as an ARDS etiology, organ injury through congestion and hypoperfusion, leading to breakdown of barrier function such as in the gut, could allow bacterial translocation and infection, manifesting as a secondary insult that worsens the original lung injury. Third, a feed‐forward pathway involving inflammatory cell recruitment, endothelial permeability, and release of systemically circulating mediators at sites of remote organ injury could then propagate to the lungs. Though testing the individual contribution of these potential pathways on clinical outcomes is extremely complex, this framework highlights the interconnectedness of these systems and the potential value of therapies targeting central pathways of disease, particularly when applied early in illness and in enriched patient populations.

### 1.4 Phenotyping Cardiovascular Involvement in ARDS

ARDS results from a dysregulated host response to injury, critically implicating inflammation, and reverberating across several organ systems. As a clinical syndrome, it is defined by a limited number of clinical features that are identifiable but downstream of the underlying biology. Therefore, patients that fulfill the criteria may still differ remarkably in the mechanisms that underpin the disease. As evident above, ARDS has had a disappointing record of translating promising targeted therapies curbing dysregulated biology into positive clinical trials, and patient heterogeneity likely plays an important role. Several investigations, leveraging clinical and proteomic analyses, have found homogeneous subgroups or phenotypes within ARDS, with differing clinical characteristics, prognosis and, at times, heterogeneity in their response to treatments previously found to be neutral (Calfee et al. 2014; Famous et al. 2017; Sinha et al. 2018). There are only limited investigations of how these ARDS phenotypes differ in cardiovascular involvement. Analyses by our group have consistently identified two phenotypes of ARDS, termed hyper‐ and hypo‐inflammatory due to their relative differences in plasma inflammatory biomarker levels. They differ in their prevalence of vasopressor use (Famous et al. 2017), death by circulatory shock (Evrard et al. 2024), degree of myocardial injury (Sinha et al. 2021) and its association with prognosis (Sanchez et al. 2025), while severity of hypoxia is not a highly differentiating feature. An analysis of a separate set of ARDS phenotypes found the more inflamed type was treated with higher vasopressor doses and had worse pulmonary vascular and RV dysfunction (Siuba et al. 2025). These analyses suggest that phenotypes defined more proximal to biology are associated with differential cardiovascular abnormalities. However, the longitudinal stability of these differences in cardiovascular involvement by phenotype, the biological mechanisms that explain them, and whether they hold differential predictive or prognostic importance is unknown.

Due to the many ways in which the cardiovascular system is impacted in ARDS, stemming from the complex physiologic interactions detailed above, these clustering techniques have also been applied to cardiovascular parameters to find homogeneous subgroups. They attempt to organize a dizzying array of abnormalities, many of which have been separately associated with clinical outcomes. In an analysis of 360 intubated patients with septic shock, investigators performed hierarchical clustering of a dozen hemodynamic and echocardiographic variables (Geri et al. 2019). The variables captured LV and RV function, cardiac output, cardiac preload, and vasopressor dosage. Their cohort was divided into 5 clusters differing primarily along type and degree of ventricular dysfunction and preload. The clusters had divergent clinical outcomes, and those treated with the highest vasopressor doses and with the highest mortality had worse LV or RV function. In an analysis of 305 mechanically ventilated patients with COVID‐19 pneumonia, using latent class analysis of 8 echocardiographic variables capturing RV and LV size and function, cardiac preload, and vasopressor dose, investigators identified 3 classes of patients (Chotalia et al. 2022). These classes differed mainly in severity of RV dysfunction and enlargement, which correlated with higher pulmonary arterial pressure, higher vasopressor dose intensity, worse respiratory compliance, and dead space ventilation, as well as receipt of renal replacement therapy and mortality. Lastly, in a cohort of 801 patients with non‐COVID ARDS, the same investigators again applied latent class analysis to a similar set of echocardiographic variables and found 4 classes of patients (Chotalia et al. 2023). Compared to their previous analysis, this one included a class with hyperdynamic LV ejection fraction, high cardiac index, and normal RV function, which rivaled the class with worse RV function for highest mortality and vasopressor requirements. This finding potentially suggested that unlike COVID‐19 pneumonia, non‐COVID ARDS had a significant number of patients with sepsis reflected in hyperdynamic LV function and vasoplegia. These analyses substantiate the usefulness of a hemodynamic phenotyping schema for understanding ARDS outcomes. The mechanisms that explain these phenotypes and whether their targeted therapy is associated with improved outcomes need to be evaluated.

## 2 Conclusions

Vascular injury, permeability, and dysfunction along pulmonary and systemic vascular beds are cornerstones of ARDS biology. This biology translates into common and significant cardiovascular derangements, including pulmonary vascular dysfunction, cardiac dysfunction, and shock, and these are associated with worse mortality. Decades of encouraging studies targeting known mediators of disease in ARDS in basic and translational models have been met with repeated disappointments upon bedside testing. However, efforts at identifying homogeneous subgroups of ARDS patients with shared biology may hold the key to devising, even invigorating re‐testing for targeted therapies and improving outcomes. Given the centrality of cardiovascular abnormalities to ARDS physiology, careful testing of these pathways in enriched populations may be especially productive.

## Author Contributions

P.A.S. and M.A.M. conducted the literature search and designed the review. P.A.S. wrote the original draft of the manuscript. P.A.S., C.O.B., M.L., C.S.C., and M.A.M. critically reviewed and revised the manuscript. All authors approved the final manuscript.

## Funding

This work was supported by funding from the NIH National Heart Lung and Blood Institute (P.A.S.: F32HL160103, L30HL165589 and C.S.C.: R35HL177135), Department of Defense, and National Institute of Allergy and Infectious Diseases (M.A.M.).

## Conflicts of Interest

The authors declare no conflicts of interest.

## Data Availability

Data sharing not applicable to this article as no datasets were generated or analysed during the current study.
